# Amino-Terminal Microdeletion within the *CNTNAP2* Gene Associated with Variable Expressivity of Speech Delay

**DOI:** 10.1155/2012/172408

**Published:** 2012-05-22

**Authors:** Amel Al-Murrani, Fern Ashton, Salim Aftimos, Alice M. George, Donald R. Love

**Affiliations:** ^1^Diagnostic Genetics, LabPlus, Auckland City Hospital, P.O. Box 110031, Auckland 1148, New Zealand; ^2^Northern Regional Genetic Service, Auckland City Hospital, Private Bag 92024, Auckland 1142, New Zealand; ^3^School of Biological Sciences, University of Auckland, Private Bag 92019, Auckland 1142, New Zealand

## Abstract

The *contactin-associated protein-like 2* (*CNTNAP2*) gene is highly expressed in the frontal lobe circuits in the developing human brain. Mutations in this gene have been associated with several neurodevelopmental disorders such as autism and specific language impairment. Here we describe a 450 kb deletion within the *CNTNAP2* gene that is maternally inherited in two male siblings, but with a variable clinical phenotype. This variability is described in the context of a limited number of other cases reported in the literature. The in-frame intragenic deletion removes a critical domain of the CNTNAP2 protein, and this case also highlights the challenges of correlating genotype and phenotype.

## 1. Introduction

The *CNTNAP2* gene encodes for Caspr2 (contactin-association protein 2), which is a member of the neurexin superfamily of transmembrane proteins that mediate cell-cell interaction in the nervous system [1]. Caspr2 is predominantly expressed in the developing human brain: in the frontal and temporal lobes, as well as in striatal circuits and in the frontal cortex of the adult brain [2]. In humans, these regions are involved in higher cortical functions, including language. Caspr2 is also essential for the localization of voltage-activated potassium channels in the juxtaparanodal region of axons, which may function to stabilize conduction and help to maintain the intermodal resting potential [1].

Several lines of evidence have identified *CNTNAP2* as an autism-susceptibility gene [2–5]. An association has been found between risk alleles in the *CNTNAP2* gene and increased local and reduced long-range frontal lobed connectivity detected by functional neuroimaging [2]. This reorganization of the frontal lobed connectivity is consistent with the abnormalities observed in both autistic patients and patients with specific language impairment. A statistically significant association has been identified between *CNTNAP2* gene variants and variation in the age of the first spoken word in autistic patients [3, 6]. In addition, rare recessive mutations in the *CNTNAP2* gene appear to segregate with seizures, language regression, and autism in some Amish pedigrees [7, 8], and to be associated with Pitt-Hopkins-like mental retardation [9]. Finally, translocation events [10, 11], partial and whole gene deletions [12–14], and microdeletions [15, 16] have been detected in the *CNTNAP2* gene. In terms of the latter, patients present with a wide spectrum of neuropsychiatric disorders, which encompass language delay, autistic spectrum disorder, epilepsy, and schizophrenia. Here we report an amino-terminal deletion within the *CNTNAP2* gene, which is maternally inherited, but is associated with a variable phenotype of speech delay. This case contrasts with the more severe clinical phenotypes detected in other rare cases with deletions that lie in this region of the *CNTNAP2* gene.

## 2. Clinical Report

The proband is a 3-year-old boy who was referred to the genetic service for an opinion regarding an abnormal molecular karyotype result. He is the second child born to a nonconsanguineous European couple with normal language development. The proband's perinatal history was uneventful and he was born at term. He exhibited mild motor delay in that he walked at around 18–20 months of age, and his speech was late to develop. At 2.5 years of age he was able to pronounce six words. At 3 years of age, he was using up to 3 words in sentences but his speech was difficult to understand. His speech therapist noted the presence of reduced tone of his orofacial musculature with associated tendency to dribble. The communication difficulties have resulted in behavioural problems. He was a nondysmorphic child with normal growth. Audiology assessment revealed normal hearing.

His 5-year-old brother exhibits normal language development for his age but he has been noted by his kindergarten teachers to have difficulties in comprehension and following instructions.

The proband's mother was not affected by any learning or speech difficulties.

### 2.1. Chromosome Microarray Analysis

Genomic DNA was isolated from peripheral blood of the proband, together with the proband's brother, mother and father using the Gentra Puregene blood kit according to the manufacturer's instructions (Qiagen Pty Ltd, MD, USA). 0.1 micrograms of genomic DNA was labelled using the Affymetrix Cytogenetics Reagent Kit and labelled DNA was applied to an Affymetrix Cytogenetics Array (2.7 million probes) according to the manufacturer's instructions (Affymetrix Inc, CA, USA). The array was scanned and the data analysed using the Affymetrix Chromosome Analysis Suite (ChAS; version 1.0.1) and interpreted with the aid of the UCSC genome browser (http://genome.ucsc.edu/; hg18 assembly).

**Figure 1:**
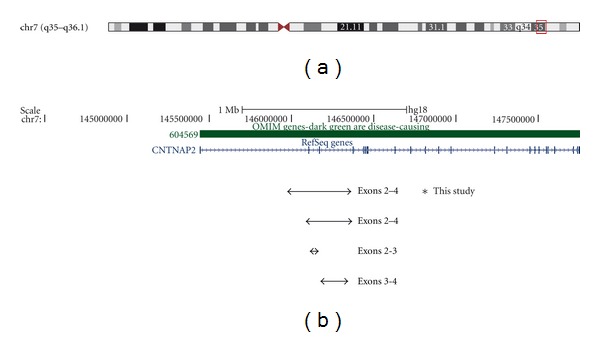
Schematic of the chromosome 7 region encompassing the *CNTNAP2* gene. (a) shows an ideogram of chromosome 7, together with the location of the *CNTNAP2* gene. (b) shows the exonic organisation of the *CNTNAP2* gene and the location and extent of the deletion detected in the proband (indicated by an asterisk), his brother and his mother, together with deletions reported by Gregor et al. [15] (patients reported as C5 and C6 with deletions of exons 2-3 and 3-4, resp.), and Mefford et al. [16] (patient reported as K034 with a deletion of exons 2–4; coordinates taken from Figure 2 of this publication), which lie in the same region of the *CNTNAP2* gene. The images presented here are taken from the UCSC genome browser (http://genome.ucsc.edu/).

**Figure 2:**
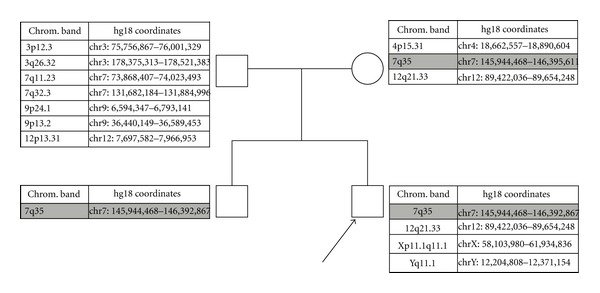
Pedigree of proband and copy number changes. The copy number changes detected in all members of the pedigree are shown; the common deletion detected in the *CNTNAP2* gene is shown in grey, and the proband is indicated by an arrow.

The proband's molecular karyotype revealed a 451 kb interstitial deletion on chromosome 7q35 (145,944,468-146,395,611); Figure 1. A similar deletion was identified in the proband's mother and brother; Figure 2. We considered that validation by quantitative real-time PCR, fluorescence *in situ* hybridization, or junctional fragment sequencing was not warranted given the array probe density over this region of chromosome 7 and the detection of this novel deletion with approximately the same coordinates in first degree relatives of the proband.

## 3. Discussion

We describe here the phenotype and molecular finding of a family (mother, father and two male siblings) with a maternally inherited chromosomal microdeletion in 7q35 within the *CNTNAP2* gene in the two siblings, formally designated as c.98_550del, p.Gln33_Trp184delinsArg in NM_014141.5 (*CNTNAP2* gene RefSeq accession number) (exons 2–4 inclusive). Critically, our case exhibits a much milder phenotype compared to three others that have been described in the literature with deletions that are encompassed by our patient's deletion. Gregor et al. [15] identified two patients with a heterozygous deletions of either exons 2-3 or 3-4. The expected effect of these deletions is a truncated CNTNAP2 protein (p.Gln33ArgfsX7) or one with an in-frame deletion (p.Gly72_Ala185del), respectively. In each case, the deletions were maternally inherited, and both manifested profound intellectual disability, absent speech, and a number of facial dysmorphic features. In the case reported by Mefford et al. [16], the patient exhibited neonatal convulsions, but no reported speech delay. The paternally inherited *CNTNAP2* gene deletion removed exons 2–4 (inclusive), and this patient also carried an interstitial 370 kb deletion on chromosome 17p13.1. The deduced breakpoints for the *CNTNAP2* gene deletion detected for this patient appear to be different from the case reported here, but the functional consequence in terms of the CNTNAP2 protein would be expected to be the same. The lack of segmental duplications in this region of the *CNTNAP2* gene suggests that simple nonallelic homologous recombination may not be the mechanism underlying the deletion events.

Taken together, the data highlights the broad spectrum of clinical manifestation associated with amino-terminal deletions that lie in the *CNTNAP2* gene. Critically, the region of the CNTNAP2 protein that is deleted in our patient encompasses the F5/8 type C, or C2-like, domain, which is defined by amino acids 33–181 (also known as the discoidin domain; http://www.uniprot.org/uniprot/Q9UHC6). This domain is likely to be functionally important as proteins carrying this domain have been implicated in cell-adhesion or developmental processes [17].

It is tempting to suggest a causal relationship between the possible haploinsufficiency in terms of expression of CNTNAP2, and speech delay, at least for the case reported here. Interestingly, Vernes et al. [18] have suggested that *CNTNAP2* gene variants may represent susceptibility factors for language-related deficits in both specific language impairment and autism.

Of importance, however, is that while the two siblings carry the same deletion in the *CNTNAP2 *gene, their clinical presentation is variable. This variability could be explained either by the two hit epistatic model [19], or allele-specific expression [20, 21]. In the case of the former, it has been suggested that two copy number variant (CNV) affecting the same functional pathway can result in a clinical phenotype that is more severe than either CNV alone. It is possible that the index case reported here has an additional CNV, or a mutation below our detection threshold and, therefore, has a more severe phenotype than his brother.

In terms of allele-specific expression, a recent two-stage genetic study identified a common polymorphism in the *CNTNAP2* gene, which is a disease variant only when inherited through the female germline [4]. Although these workers did not give a reason for this parent-of-origin bias, their finding is compelling due to the fact that this parent-of-origin and gender effect reiterates the pattern of autism inheritance. A more recent study of autistic children and their parents described a case of a maternally transmitted deletion in the promoter region of the *CNTNAP2* gene [20]. The expression of the *CNTNAP2* gene transcript was significantly decreased compared to the wild type father and control; interestingly, the expression in the mother was also decreased but not as significantly as the index case. In contrast to the two-hit model, allele-specific expression may control penetrance and explain why some carriers are unaffected [20, 21].
